# Polygenic risk score adds to a clinical risk score in the prediction of cardiovascular disease in a clinical setting

**DOI:** 10.1093/eurheartj/ehae342

**Published:** 2024-06-07

**Authors:** Nilesh J Samani, Emma Beeston, Chris Greengrass, Fernando Riveros-McKay, Radoslaw Debiec, Daniel Lawday, Qingning Wang, Charley A Budgeon, Peter S Braund, Richard Bramley, Shireen Kharodia, Michelle Newton, Andrea Marshall, Andre Krzeminski, Azhar Zafar, Anuj Chahal, Amadeeep Heer, Kamlesh Khunti, Nitin Joshi, Mayur Lakhani, Azhar Farooqi, Vincent Plagnol, Peter Donnelly, Michael E Weale, Christopher P Nelson

**Affiliations:** Department of Cardiovascular Sciences, University of Leicester, BHF Cardiovascular Research Centre, Glenfield Hospital, Groby Road, Leicester LE3 9QP, UK; NIHR Leicester Biomedical Research Centre, Glenfield Hospital, Groby Road, Leicester LE3 9QP, UK; Department of Cardiovascular Sciences, University of Leicester, BHF Cardiovascular Research Centre, Glenfield Hospital, Groby Road, Leicester LE3 9QP, UK; NIHR Leicester Biomedical Research Centre, Glenfield Hospital, Groby Road, Leicester LE3 9QP, UK; Department of Cardiovascular Sciences, University of Leicester, BHF Cardiovascular Research Centre, Glenfield Hospital, Groby Road, Leicester LE3 9QP, UK; NIHR Leicester Biomedical Research Centre, Glenfield Hospital, Groby Road, Leicester LE3 9QP, UK; Genomics plc, King Charles House, Park End Street, Oxford OX1 1 JD, UK; Department of Cardiovascular Sciences, University of Leicester, BHF Cardiovascular Research Centre, Glenfield Hospital, Groby Road, Leicester LE3 9QP, UK; NIHR Leicester Biomedical Research Centre, Glenfield Hospital, Groby Road, Leicester LE3 9QP, UK; Department of Cardiovascular Sciences, University of Leicester, BHF Cardiovascular Research Centre, Glenfield Hospital, Groby Road, Leicester LE3 9QP, UK; NIHR Leicester Biomedical Research Centre, Glenfield Hospital, Groby Road, Leicester LE3 9QP, UK; Department of Cardiovascular Sciences, University of Leicester, BHF Cardiovascular Research Centre, Glenfield Hospital, Groby Road, Leicester LE3 9QP, UK; NIHR Leicester Biomedical Research Centre, Glenfield Hospital, Groby Road, Leicester LE3 9QP, UK; Department of Cardiovascular Sciences, University of Leicester, BHF Cardiovascular Research Centre, Glenfield Hospital, Groby Road, Leicester LE3 9QP, UK; NIHR Leicester Biomedical Research Centre, Glenfield Hospital, Groby Road, Leicester LE3 9QP, UK; School of Population and Global Health, University of Western Australia, Perth WA 6009, Australia; Department of Cardiovascular Sciences, University of Leicester, BHF Cardiovascular Research Centre, Glenfield Hospital, Groby Road, Leicester LE3 9QP, UK; NIHR Leicester Biomedical Research Centre, Glenfield Hospital, Groby Road, Leicester LE3 9QP, UK; Department of Cardiovascular Sciences, University of Leicester, BHF Cardiovascular Research Centre, Glenfield Hospital, Groby Road, Leicester LE3 9QP, UK; NIHR Leicester Biomedical Research Centre, Glenfield Hospital, Groby Road, Leicester LE3 9QP, UK; Department of Cardiovascular Sciences, University of Leicester, BHF Cardiovascular Research Centre, Glenfield Hospital, Groby Road, Leicester LE3 9QP, UK; NIHR Leicester Biomedical Research Centre, Glenfield Hospital, Groby Road, Leicester LE3 9QP, UK; Department of Cardiovascular Sciences, University of Leicester, BHF Cardiovascular Research Centre, Glenfield Hospital, Groby Road, Leicester LE3 9QP, UK; NIHR Leicester Biomedical Research Centre, Glenfield Hospital, Groby Road, Leicester LE3 9QP, UK; Department of Cardiovascular Sciences, University of Leicester, BHF Cardiovascular Research Centre, Glenfield Hospital, Groby Road, Leicester LE3 9QP, UK; NIHR Leicester Biomedical Research Centre, Glenfield Hospital, Groby Road, Leicester LE3 9QP, UK; Albany House Medical Centre, Wellingborough NN8 4RW, UK; Diabetes Research Centre, University of Leicester, Leicester General Hospital, Leicester LE5 4PW, UK; Diabetes and Cardiovascular Medicine General Practice Alliance Federation Research and Training Academy, Northampton NN2 6AL, UK; South Leicestershire Medical Group, Kibworth Beauchamp LE8 0LG, UK; Lakeside Healthcare, Corby NN17 2UR, UK; NIHR Leicester Biomedical Research Centre, Glenfield Hospital, Groby Road, Leicester LE3 9QP, UK; Diabetes Research Centre, University of Leicester, Leicester General Hospital, Leicester LE5 4PW, UK; Willowbrook Medical Centre, Leicester LE5 2NL, UK; Department of Health Sciences, University of Leicester, Leicester LE1 7RH, UK; Department of Health Sciences, University of Leicester, Leicester LE1 7RH, UK; Genomics plc, King Charles House, Park End Street, Oxford OX1 1 JD, UK; Genomics plc, King Charles House, Park End Street, Oxford OX1 1 JD, UK; Genomics plc, King Charles House, Park End Street, Oxford OX1 1 JD, UK; Department of Cardiovascular Sciences, University of Leicester, BHF Cardiovascular Research Centre, Glenfield Hospital, Groby Road, Leicester LE3 9QP, UK; NIHR Leicester Biomedical Research Centre, Glenfield Hospital, Groby Road, Leicester LE3 9QP, UK

**Keywords:** CVD risk assessment, Polygenic risk scores, QRISK2, SCORE2, ASCVD-PCE

## Abstract

**Background and Aims:**

A cardiovascular disease polygenic risk score (CVD-PRS) can stratify individuals into different categories of cardiovascular risk, but whether the addition of a CVD-PRS to clinical risk scores improves the identification of individuals at increased risk in a real-world clinical setting is unknown.

**Methods:**

The Genetics and the Vascular Health Check Study (GENVASC) was embedded within the UK National Health Service Health Check (NHSHC) programme which invites individuals between 40–74 years of age without known CVD to attend an assessment in a UK general practice where CVD risk factors are measured and a CVD risk score (QRISK2) is calculated. Between 2012–2020, 44,141 individuals (55.7% females, 15.8% non-white) who attended an NHSHC in 147 participating practices across two counties in England were recruited and followed. When 195 individuals (cases) had suffered a major CVD event (CVD death, myocardial infarction or acute coronary syndrome, coronary revascularisation, stroke), 396 propensity-matched controls with a similar risk profile were identified, and a nested case-control genetic study undertaken to see if the addition of a CVD-PRS to QRISK2 in the form of an integrated risk tool (IRT) combined with QRISK2 would have identified more individuals at the time of their NHSHC as at high risk (QRISK2 10-year CVD risk of ≥10%), compared with QRISK2 alone.

**Results:**

The distribution of the standardised CVD-PRS was significantly different in cases compared with controls (cases mean score .32; controls, −.18, *P* = 8.28×10^−9^). QRISK2 identified 61.5% (95% confidence interval [CI]: 54.3%–68.4%) of individuals who subsequently developed a major CVD event as being at high risk at their NHSHC, while the combination of QRISK2 and IRT identified 68.7% (95% CI: 61.7%–75.2%), a relative increase of 11.7% (*P* = 1×10^−4^). The odds ratio (OR) of being up-classified was 2.41 (95% CI: 1.03–5.64, *P* = .031) for cases compared with controls. In individuals aged 40–54 years, QRISK2 identified 26.0% (95% CI: 16.5%–37.6%) of those who developed a major CVD event, while the combination of QRISK2 and IRT identified 38.4% (95% CI: 27.2%–50.5%), indicating a stronger relative increase of 47.7% in the younger age group (*P* = .001). The combination of QRISK2 and IRT increased the proportion of additional cases identified similarly in women as in men, and in non-white ethnicities compared with white ethnicity. The findings were similar when the CVD-PRS was added to the atherosclerotic cardiovascular disease pooled cohort equations (ASCVD-PCE) or SCORE2 clinical scores.

**Conclusions:**

In a clinical setting, the addition of genetic information to clinical risk assessment significantly improved the identification of individuals who went on to have a major CVD event as being at high risk, especially among younger individuals. The findings provide important real-world evidence of the potential value of implementing a CVD-PRS into health systems.

## Structured Graphical Abstract

**Structured Graphical Abstract:**
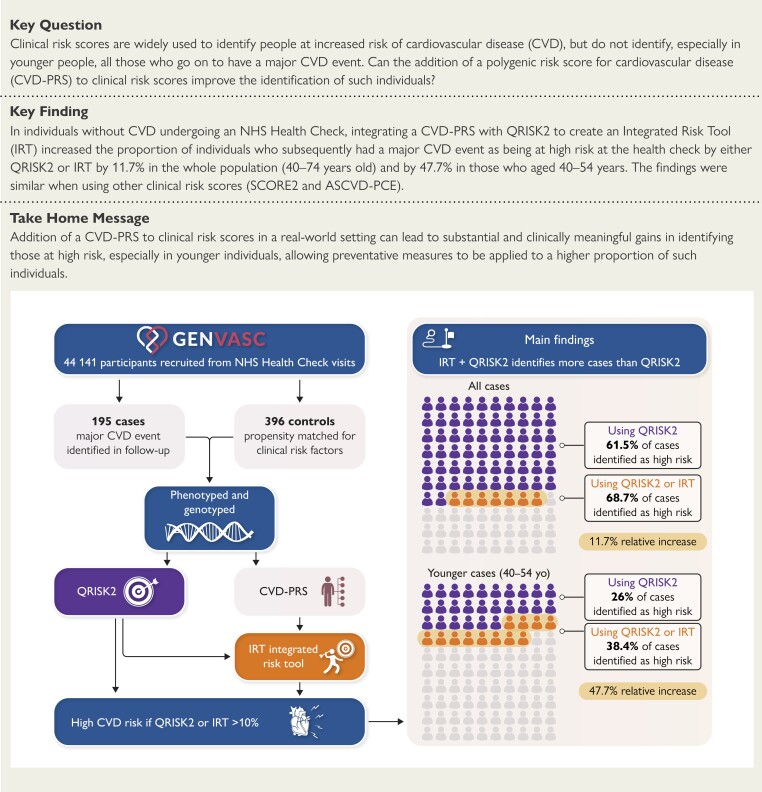
GENVASC design and key findings: The GENVASC study was set up to evaluate whether the addition of a polygenic risk score for cardiovascular disease (CVD-PRS) to clinical risk assessment at an NHS Health Check would increase the number of individuals who went to have a major CVD event as being at high risk at their health check. 44 141 individuals free of CVD were recruited at their NHS Health Check and when 195 CVD events had occurred during follow-up, a retrospective case-control study was undertaken to see how many additional individuals would have been identified as high risk at their health check by the addition of the CVD-PRS in the form of an integrated risk tool (IRT) to assessment by the clinical risk score (QRISK2) alone.

**See the editorial comment for this article ‘Polygenic scores in real-world cardiovascular risk prediction: the path forward for assessing worth?', by V. Pillutla and K.G. Aragam, https://doi.org/10.1093/eurheartj/ehae442.**

## Introduction

In the last 15 years, hundreds of genetic variants that affect the risk of major cardiovascular diseases (CVD) such as coronary artery disease (CAD) and stroke have been identified through genome-wide association studies (GWAS).^1,2^ The incremental risk associated with each variant is small (typically <10%), but studies have shown that when the variants are combined into a polygenic risk score (PRS), such genetic information can partition patients into different trajectories of subsequent CVD risk.^1,2^ Furthermore, several studies have shown that a PRS for CVD (or CAD) adds to risk assessment based on clinical risk scores such as the Framingham, QRISK and atherosclerotic cardiovascular disease pooled cohort equations (ASCVD-PCE) scores.^3–17^ These findings have prompted an evaluation of the possible introduction of such risk scores into clinical practice.^18^ However, most of the evidence for the potential value of PRS for CVD has been generated in research cohorts that may not be representative of the general population, and the potential value in a clinical setting, and how much it adds to clinical risk assessment, has not been extensively examined.

In 2008, the UK government introduced a new NHS Health Check (NHSHC) to identify those at increased risk of CVD.^19^ Implemented through primary care general practices (GPs), the NHSHC invites individuals aged 40–74 years without known CVD to attend for an assessment where CVD risk factors including blood pressure, body mass index and lipid profiles are measured^19^ and a QRISK2 (now QRISK3) score^20^ calculated. Those categorised at high (≥10%) 10-year risk are then given tailored management, including lifestyle advice and the prescription of statins and anti-hypertensive medication, if not already on such medications. The NHSHC programme provides an ideal setting to assess the potential clinical value of a PRS for CVD in a real-world setting and quantify the extent to which it classifies individuals as being at higher risk when added to a clinical risk score and thus makes them more ‘visible’ for advice and intervention. We established the Genetics and the Vascular Health Check Study (GENVASC) within the NHSHC framework to examine this question and here report the findings from a nested genetic case-control study of the value of a PRS for CVD within the cohort.

## Methods

### Genetics and vascular health check study (GENVASC)

GENVASC was initiated in September 2012. The study recruited patients attending an NHSHC in one of 147 GPs in two counties in England (Leicestershire and Northamptonshire). The study was approved by the UK National Research Ethics Service East Midlands Committee (Approval No 12/EM/0208) and all participants provided written consent to participate. Blood samples for research including genetic analysis were collected at the time that blood was drawn for clinical blood tests related to the NHSHC. Relevant clinical information including measurements (blood pressure, lipid profile, body mass index, and calculated QRISK2 score) carried out at the health check were downloaded from the GP records electronically. Subsequently, further downloads were made of GP records every 6 months and integrated with downloads of hospital records to identify new cardiovascular outcomes (cardiovascular death, myocardial infarction [MI] or acute coronary syndrome [ACS], coronary revascularisation via percutaneous coronary intervention [PCI] or coronary artery bypass graft [CABG] surgery), and stroke. The cause of death was ascertained from primary and secondary care records and death certificates. At the closure of recruitment in GENVASC in October 2020, 44,141 participants had been recruited.

### Nested case-control genetic study

In late 2019, when over 27 000 participants had been recruited and around 200 major CVD outcomes had occurred, we designed a nested-case control study to examine the incremental value of a CVD-PRS in identifying those who suffered an event. Major CVD cases were identified on a hierarchical basis of CVD death, MI/ACS, PCI/CABG, and stroke. To reduce potential confounding, propensity score analyses using age, sex, ethnicity, smoking status, body mass index, and QRISK2 score were used to identify controls without replacement in an approximate 2:1 ratio to the cases. Adequate covariate balance between the cases and controls was assessed using standardised mean differences (SMD).^21^ After genotype quality control (see below), there were 195 cases with major CVD events and 396 controls.

### Genotyping and imputation

DNA was extracted from venous blood samples collected in ethylenediaminetetraacetic acid, using QIAsymphony DSP DNA kits (Qiagen, UK) and quality was checked using 260/280 nm and 260/230 nm absorbance ratios. Genotyping was undertaken using the Thermo Fisher UK Biobank Axiom™ array (Thermo Fisher Scientific, Waltham, MA, USA, Product No 902502). The genotypes underwent quality control filtering prior to imputation. Single nucleotide polymoprhisms (SNPs) with a call rate < 98% were excluded as were variants where the minor allele frequency was < .005 or if Hardy Weinberg Equilibrium deviation was *P* < 1×10^−06^, providing information on average on 641 038 variants. Two cases were excluded because of failed genotyping.

Imputation was undertaken on the Sanger Imputation Service (https://www.sanger.ac.uk/tool/sanger-imputation-service/) with SHAPEIT for pre-phasing^22^ and Positional Burrows Wheeler Transform^23^ for imputation using the Haplotype Reference Consortium release 1.1 reference panel^24^ and the UK10K + 1000 Genomes phase 3 reference panel.^25^ Post-imputation the two reference panels were combined using QCTOOLv2 (https://www.well.ox.ac.uk/∼gav/qctool_v2/), providing information on up to 92, 618 227 variants.

### Polygenic risk score and integrated risk tool

Details of the derivation and validation of the CVD-PRS used in this study (independently of GENVASC) and its integration with clinical risk scores to create an integrated risk tool (IRT) have been described previously.^11^ Briefly, ten GWAS datasets for different atherosclerotic CVDs (including MI and stroke), representing individuals from multiple ancestry groups and from different geographies, were meta-analysed to derive the CVD-PRS. LDpred^26^ was used to derive a set of PRS weights from the meta-analysis dataset described above, using priors incorporating functional information following the method of Marquez-Luna et al.^27^ The CVD-PRS contained 2 829 817 non-zero SNP weights spread throughout the genome. The CVD-PRS was corrected for ancestry via a principal component analysis-based approach, which standardises the scores to approximately zero mean and unit variance using metrics obtained from an external reference dataset (1000 genomes reference panel). An additional four cohorts were used to train the CVD-PRS effect size. A logistic function was used to combine the CVD-PRS with the PRS effect size and the person's QRISK2 score^20^ (or for comparative analyses, their ASCVD-PCE^28^ or SCORE2^29^ scores), to generate the IRT score for 10-year risk of CVD event based on both QRISK2 (or ASCVD-PCE or SCORE2) and genetic information, as described previously.^11^ To assess the additional value of the IRT, we calculated the difference in the proportion of cases that would have been identified as high risk at the NHSHC by a combination (high risk by one or the other) of IRT and QRISK2 (or ASCVD-PCE or SCORE2), over those identified by the respective clinical risk scores alone. To define high risk (actionable according to relevant guidelines), we used ≥10% for QRISK2 and its IRT, 7.5% for PCE and its IRT, and 2.5%–7.5% (depending on age) for SCORE2 and its IRT, as per UK, US and European guidelines, respectively.^30–32^ To generate CIs and *P*-values for differences in sensitivity between the clinical scores and the combination of IRT with the clinical scores QRISK2, we employed the R package ‘DTComPair’ using the standard Wald interval calculated from the sampling error of the difference. We assessed the differences for all cases and also sub-groups based on gender, age and ethnicity. Due to the use of matched controls in this study, full net reclassification improvement metrics (summarising reclassification in both cases and controls) are not presented but we provide a case net reclassification index (case-NRI).

## Results

### Subjects

Key characteristics of the cases and controls are shown in *Table 1*. The cases and controls did not significantly differ in their clinical characteristics and adequate covariate balance was achieved across all clinical characteristics used in the propensity score analysis (all SMD < .1). Subjects were on average 57.5 years of age at the time of their enrolment, 62% were male and 76.5% were of white ethnicity. The non-white subjects were predominantly of South Asian (Indian/Pakistani) origin. The average QRISK2 score was 12.6 (SD 7.3) % for cases and 11.6 (SD 7.7) % for controls. In a hierarchical order of inclusion, 5.6% (*n* = 11) of cases were included due to a CVD death, 57.9% (*n* = 113) due to an MI/ACS, 12.8% (*n* = 25) because of a coronary intervention (PCI/CABG), and 23.5% (*n* = 46) due to a stroke of which 40 were ischaemic and the rest were indeterminate. 11.3% of cases and 10.1% of controls were already taking a statin at the time of their health check.

**Table 1:** Characteristics of cases and controls at baseline

	Cases		Controls		*P*-value
	*N* = 195		*N* = 396		
Age, years	57.3	(9.2)	57.7	(9.6)	.674
Age 40–54	73	(37.4)	142	(35.9)	.777
Male gender	126	(64.6)	240	(60.6)	.252
White ethnicity	151	(77.4)	302	(76.3)	.700
Smoking status					
Current smoker	67	(34.4)	116	(29.3)	.322
Ex-smoker	39	(20.0)	74	(18.7)	
Never smoker	89	(45.6)	206	(52.0)	
Systolic blood pressure, mm Hg	134.4	(16.3)	132.6	(15.7)	.185
Diastolic blood pressure, mm Hg	81.5	(9.7)	80.3	(10.6)	.191
Body mass index, kg/m^2^	27.8	(5.8)	28.0	(5.1)	.718
Diabetes	6	(3.1)	8	(2.0)	.294
Statins, prior to NHSHC	22	(11.3)	40	(10.1)	.584
Lipids					
Total-cholesterol, mmol/L	5.6	(1.1)	5.5	(1.1)	.180
LDL-cholesterol, mmol/L	3.4	(.9)	3.3	(.9)	.239
HDL-cholesterol, mmol/L	1.4	(.4)	1.4	(.4)	.167
Triglycerides, mmol/L	1.8	(1.3)	1.7	(1.0)	.719
Total cholesterol: HDL ratio	4.4	(1.4)	4.1	(1.2)	.011
Average QRISK2 score	12.6	(7.3)	11.6	(7.7)	.171
QRISK2 grouping					
High risk (≥10)	120	(61.5)	209	(52.8)	.128
Intermediate risk (5–10)	42	(21.5)	102	(25.8)	
Low risk (<5)	33	(16.9)	85	(21.4)	

Quantitative (continuous) values are presented as mean (standard deviation) while categorical values are presented as number (percentage).

CVD, cardiovascular disease; MI, myocardial infarction; ACS, acute coronary syndrome; PCI, percutaneous coronary intervention; CABG, coronary artery bypass surgery.

*P*-values (*α* = .05) are from Student's *t*-test for continuous data and a χ^2^ test for categorical data.

For a proportion of participating practices, we had information on the total number of NHSHCs undertaken annually during the recruitment period for GENVASC. Across these practices, on average 31.7% of subjects having an NHSHC were recruited into GENVASC (see Supplementary data online, *Figure S1*). Compared with a broader group of contemporary subjects undergoing NHSHC across England as reported by Patel et al.,^33^ GENVASC participants had similar sex and age distributions but, reflecting the socio-demography of the UK East Midlands population where GENVASC participants were recruited, there were a higher proportion of South Asians and a lower number of subjects with higher Townsend Deprivation scores, a marker of social deprivation with higher scores representing greater social deprivation (see Supplementary data online, *Table S1*).

### Polygenic risk score

**Figure 1:**
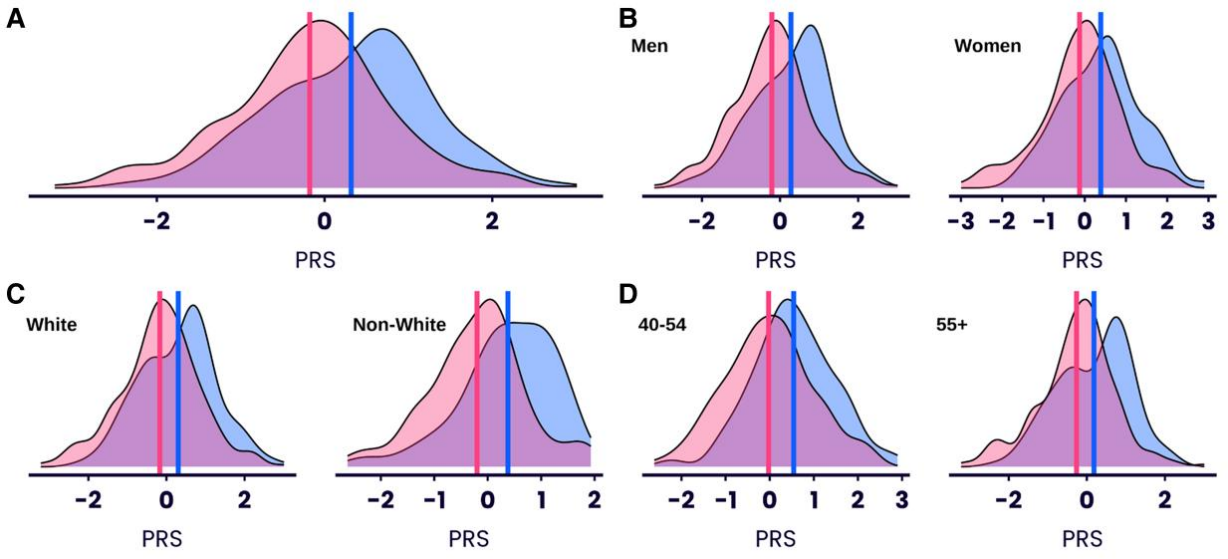
Distribution of standardised cardiovascular disease polygenic risk score by case status. (*A*) Distribution of cardiovascular disease polygenic risk score in all major cardiovascular disease event cases (blue) and controls (pink); (*B*) Distribution of cardiovascular disease polygenic risk score in major cardiovascular disease event cases (blue) and controls (pink) partitioned by gender; (*C*) Distribution of cardiovascular disease polygenic risk score in major cardiovascular disease event cases (blue) and controls (pink) partitioned by ethnicity. (*D*) Distribution of cardiovascular disease polygenic risk score in major cardiovascular disease event cases (blue) and controls (pink) partitioned by age group. Vertical lines indicate mean polygenic risk score value in each category

**Figure 2:**
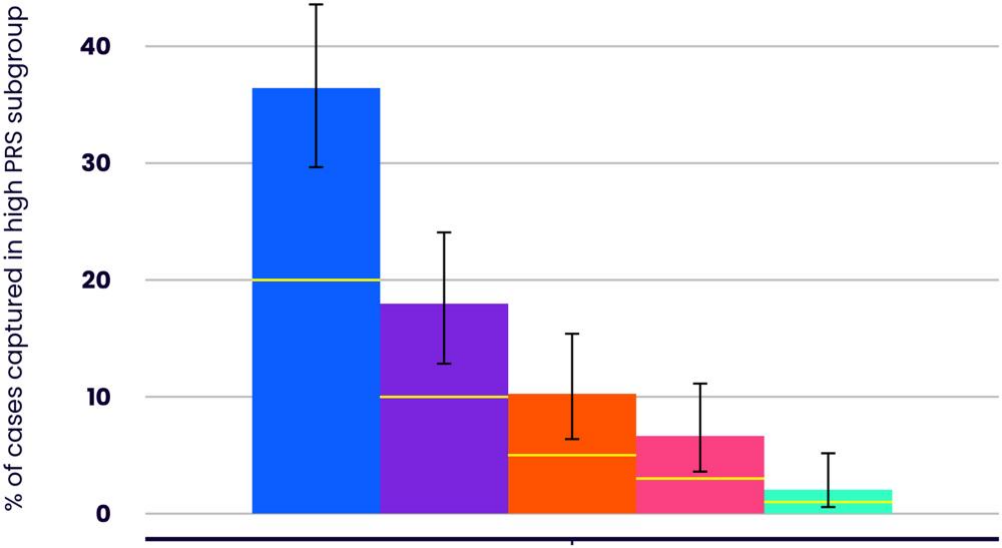
Enrichment of cases in the top percentiles of the cardiovascular disease polygenic risk score distribution. Bars represent the top 20% (blue), top 10% (purple), top 5% (orange), top 3% (pink) and top 1% (green) of the polygenic risk score distribution. *Y*-axis represents the proportion of major cardiovascular disease event cases captured in individuals at or above the stated polygenic risk score percentile. Yellow lines indicate no-enrichment values (% of cases matches % of polygenic risk score distribution)

The distributions of the standardised CVD-PRS for cases and controls are shown in *Figure 1A*. The mean normalised CVD-PRS score for cases (.32) was significantly higher than that of controls (−.18) (*P* = 8.3×10^−9^). CVD-PRS performed equally well in differentiating male (cases, .28, control, −.21 mean PRS, *P* = 7.7×10^−6^) and female (cases .39; control, −.12, *P* = 2.3×10^−4^) cases from their respective controls (*Figure 1B*). The differentiation between cases and controls was not significantly different between males and females (*P* = .89). Similarly, the distributions of CVD-PRS for cases and controls were significantly different for both white (case mean CVD-PRS score .30, control mean PRS score −.17, *P* = 2.64×10^−6^) and non-white ethnicities (case mean CVD-PRS score .38, control mean PRS score −.20, *P* = 5.38×10^−4^) (*Figure 1C*). Again, the differentiation between cases and controls was not significantly different between white and non-white ethnic groups (*P* = .59). Splitting by age group (40–54 vs. 55+) also showed significantly higher mean CVD-PRS for cases compared with controls in both groups (40–54 age group: case mean PRS score .54, control mean PRS score −.02, *P* = 6.27×10^−5^; 55 + age group: case mean PRS score .18, control mean PRS score −.26, *P* = 2.69×10^−5^), with no significant difference between groups (*P* = .50) (*Figure 1D*). Cases with a major CVD outcome were enriched by between 1.7 and 2.2-fold in the upper percentiles of the PRS distribution than would be expected by chance (*Figure 2*).

### Incremental value of adding cardiovascular disease polygenic risk score to QRISK2 and other clinical risk scores

**Figure 3:**
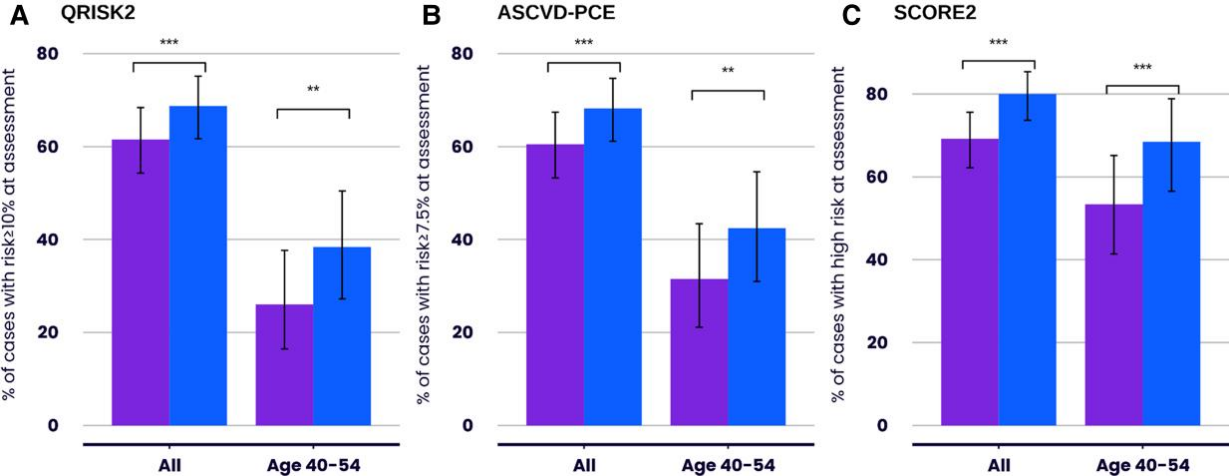
Percentage of cases identified as at high risk at the time of the National Health Service Health Check by QRISK2, Atherosclerotic Cardiovascular Disease Pooled Cohort Equations or SCORE2 alone or a combination of QRISK2, Atherosclerotic Cardiovascular Disease Pooled Cohort Equations or SCORE2 and integrated risk tool. *Y*-axis represents the percentage of major cardiovascular disease event cases identified as at high risk by QRISK2 (*A*), atherosclerotic cardiovascular disease pooled cohort equations (*B*) or SCORE2 (*C*) (purple) or a combination of each risk tool and their respective integrated risk tool (blue) at the time of their National Health Service Health Check for all cases and for those aged 40–54 years of age at the time of their assessment. ** indicates that the *P*-value for difference is <.01. *** indicates that the *P*-value for difference is <.001

QRISK2 identified 61.5% (95% CI: 54.3%–68.4%) of subjects who subsequently developed a major CVD event as being at high risk at the time of their NHSHC (*Figure 3A*). Integrating a CVD-PRS with QRISK2 to create an IRT, increased the number identified at high risk by either QRISK2 or IRT to 68.7% (95% CI: 61.7%–75.2%), an absolute increase of 7.2% (95% CI: 3.6%–10.8%) (*P* = 1×10^−4^) and a relative increase of 11.7% (*Figure 3A*). This increase was due to an additional 14/75 (18.7%) of cases with a QRISK2 < 10% being reclassified as being at high risk. All the individuals that were up-classified had an intermediate (5%–10%) QRISK2 score.

Using the alternative ASCVD-PCE and SCORE2 scores, respectively, 60.5% (95% CI: 53.3%–67.4%) and 69.2% (95% CI: 62.2%–75.6%) of those with a major CVD event would have been identified as high risk. This increased to 68.2% (95% CI: 61.2%–74.7%) and 80.0% (95% CI: 73.7%–85.4%), respectively, with relative increases of 12.7% and 15.6%, when the CVD-PRS was combined with these scores to create an IRT (*Figure 3B* and *C*).

Age is a major component of risk calculated with the conventional clinical risk scores, and therefore these tools identify proportionately fewer younger subjects above a given fixed high-risk threshold. Indeed, in our study, QRISK2 only identified 26.0% (95% CI: 16.5%–37.6%) of the 73 subjects aged between 40–54 years old who suffered a major CVD event as being at high risk at the time of their NHSHC (*Figure 3A*). The combination of QRISK2 and IRT increased this to 38.4% (95% CI: 27.2%–50.5%), an absolute increase of 12.4% and a relative increase of 47.7% (*P* = .001) (*Figure 3A*), due to up classification of an additional 9/54 (18.7%) cases who had a QRISK2 score of <10% as being high risk by the IRT. The findings were similar when using the ASCVD-PCE and SCORE 2 scores (*Figures 3B* and *C*). In additional sub-group analyses, the combination of QRISK2 and IRT significantly increased the number of women identified at high risk as well as those from non-White ethnic backgrounds (*P* = .01 and .02, respectively) (see Supplementary data online, *Figure S2*).

As a proportion of cases were taking a statin at the time of their NHSHC, in a sensitivity analysis we assessed the added value of CVD-PRS in cases with major CVD events who were not on a statin at their assessment (*n* = 173). In these subjects, the combination of QRISK2 and IRT increased the percentage of subjects defined as at high risk by an absolute 8.1% (4.0%–12.2%, *P* = 9.0×10^−5^) from 57.8% to 65.9% in all cases and by 12.5% (4.9%–20.1%, *P* = .001) from 25.0% to 37.5% in those aged 40–54 years.

To exclude the possibility that the up-classification of risk with the IRT is non-specific and would also be seen in controls, we examined up-classification rates in cases with a major CVD event, and controls, initially flagged as at low risk by QRISK2. The odds ratio (OR) of being up-classified was 2.41 (95% CI: 1.03–5.64, *P* = .031) for such cases compared with controls. This was also the case for non-white ethnicities (OR 7.97 [1.18–90.88], *P* = .015).

Although, overall, the combination of QRISK2 and IRT increased the proportion of individuals identified as being at high risk who subsequently developed a major CVD event, amongst the 61.5% of such cases identified as high risk by QRISK2, the IRT re-classified 6.1% as being at <10% 10-year risk. Therefore, if the IRT were used on its own (to both up-classify and down-classify individuals) it would have identified only 1.1% additional cases over QRISK2 (case-NRI). In the 26.0% of cases between 40–54 years of age identified as high risk by QRISK2, the IRT down-classified 2.7% as at <10%, 10-year risk. Thus, in this age-group the case-NRI was 9.7%. Note that due to the case-control design of our study, with matched controls including QRISK2 scores, we have not computed C-statistics or other measures of discrimination or calibration that are applicable to population studies, as these would be inappropriate and misleading.

## Discussion

Screening for CVD risk with clinical risk scores is now well-established in many health systems and plays an important role in reducing the burden of CVD. However, it is recognised that such scores are by no means perfect at identifying those at increased risk. Although adding PRSs for CAD or CVD has been shown to improve traditional metrics for risk prediction such as model discrimination, calibration, and net reclassification, most studies^3–17^ have reported modest overall improvements in these metrics raising questions about their clinical utility. Here, we have taken a different approach to assessing the value of adding a CVD-PRS. First, following previous work,^11^ we have combined the CVD-PRS with a clinical risk score to create an IRT. The IRT has the advantage that it returns an updated risk on the same scale, and thus the same actionable risk threshold, as the clinical risk score, which obviates the need to define a separate high-risk threshold for the CVD-PRS outside of current guidelines and therefore should be simpler for clinicians to interpret and apply findings in clinical practice. Then, in a real-world setting of a middle-aged multi-ethnic population specifically undergoing CVD risk assessment, we have evaluated, for the first time, the clinically-relevant question of whether placing an IRT alongside a clinical risk score would have identified more people who went on to have a CVD event as being at high risk at the time of their risk assessment, and therefore make them more visible for preventative measures, compared to a clinical risk score alone. We report several notable observations that are relevant to the potential inclusion of PRSs into CVD risk assessment.

Our study confirmed that current clinical risk scores only identify a proportion of individuals who subsequently go on to have a major CVD event. Overall, QRISK2, ASCVD-PCE and SCORE2 identified about three in five individuals who subsequently developed a major CVD event, using their respective definitions of high risk. For all scores, the addition of information from a CVD-PRS (as part of the IRT) increased the relative number of such cases identified as at high risk at the NHSHC by between 11.7%–15.6%, demonstrating not only a significant increase but utility across different widely-used clinical risk scores (*Structured Graphical Abstract*).

Because of the nested case-control design of our study, we are unable to directly infer the additional proportion of individuals in the population that would be up-classified by the IRT from our data, an important issue for any clinical implementation. We have previously reported that in UK Biobank an extra 4.7% of individuals were up-classified to be at high risk by either QRISK2 or the IRT compared to QRISK2 alone.^11^ However, perhaps of more direct relevance, a recent study (the HEART study^34^) also in a primary care NHS health check population which assessed the acceptance of the same IRT, found that 19.4% (161/832) of the study population were classified as high risk by QRISK2, and 24.5% (204/832) by either QRISK2 or the IRT. This indicates a direct estimated increase in up-classification to a high risk of 5.2% (43/832) in the study population, or 6.4% (43/671) amongst those in the low QRISK-2 population due to the adoption of the IRT. Applying these findings to our study, if up-classification amongst the 38.5% of low-QRISK2 cases by IRT was random, then only 2.5% of such cases (.385 * .064) would have been up-classified. Instead, in the GENVASC study we found that 7.2% of the subsequent cases were up-classified as high risk at their NHSHC, underscoring the substantial clinical gain from the addition of the CVD-PRS to the risk assessment.

Cardiovascular disease risk thresholds are not fixed and lowering the QRISK2 threshold for what is deemed high risk would also enable additional cases to be identified. For comparison, we, therefore, examined the distribution of QRISK2 scores in our cases and found that a QRISK2 score of ≥8.3% 10-year risk would have led to an equivalent absolute uplift in the identification of cases as the addition of the IRT to the risk assessment. In the HEART study,^34^ this QRISK2 threshold would have led to 26.0% of individuals being deemed as high risk, resulting in 1.5% more individuals being considered for treatment than that from a combination of QRISK2 and/or IRT threshold of ≥10% 10-year risk.

Important questions related to the application of CVD-PRS include whether it will perform equally well in women as in men and in different ethnic groups. We found no significant difference in the performance of the CVD-PRS in women compared with men, and the IRT increased the number of women identified as at high risk to the same proportion as in men. Because the majority of underpinning GWASs have been done in individuals of white ethnicity, there has been concern about the ability of CVD-PRSs to predict risk in non-white ancestries. However, we found no significant difference in the performance of our CVD-PRS in non-white ethnicities (albeit predominantly South Asian) compared to white individuals, a finding consistent with our previous US validation study.^11^ Therefore, while further CVD-GWASs in different ethnicities will help to improve and refine PRSs and potentially allow ethnic specific CVD-PRSs to be developed,^35^ this finding indicates that the addition of CVD-PRSs into risk assessment will be valuable across ethnicities.

A major component of risk prediction in clinical risk scores is age and it is known that such scores perform less well in younger people when fixed risk thresholds are used. Indeed, in GENVASC we observed that for individuals between 40 and 54 years of age, QRISK2 and the other clinical scores only identified approximately one in four to one in two subjects who subsequently had a major CVD event. As genetic risk likely plays a greater role in causing premature CVD, it might be better at identifying younger individuals at increased risk. Consistent with this, in subjects aged 40–54 years, the combination of QRISK2 and IRT increased the proportion of individuals who subsequently developed a major CVD event, as being at high risk at their NHSHC by around 50%, an almost four-fold higher proportion compared to the overall group (*Structured Graphical Abstract*). The addition of genetic information in assessing risk may therefore be particularly valuable in younger individuals, as other studies have also indicated,^10,11,16^ although it should be noted that even with the addition of such information only around two in five individuals in this age range who developed a major event were identified as being at high risk at their health check.

Although, overall, the combination of QRISK2 and IRT identified a greater proportion of individuals who subsequently developed a CVD event as being at high risk compared with QRISK2 alone, the IRT also downgraded the risk of a proportion of individuals who were deemed to be at high risk by QRISK2, who went on to have an event. This is likely to be because such individuals carry a lower genetic risk. Therefore, while on its own the IRT identified a greater number of cases as being at higher risk at the NHSHC compared with QRISK2, especially amongst those between 40 and 54 years of age, as the main purpose of risk assessment is to identify the highest number of individuals who are truly at high risk to maximise preventive benefit, this finding suggests that in clinical application, the IRT could be used primarily to up-classify people and that individuals who are deemed to be high risk with a clinical risk score but at lower risk with the IRT should continue to be managed as at high risk. This is consistent with the recommendations of others that, in the context of a CVD risk assessment, a CVD-PRS should be primarily used as a ‘risk-enhancing factor’.^36,37^

Any benefits in the prevention of CVD events from the inclusion of a CVD-PRS to risk assessment will depend on the measures taken to reduce risk in those identified as being at high risk but should scale up proportionately for the additional proportion of individuals identified in this category, that is, by ∼12% in the whole adult population and by almost 50% in individuals aged 40–54 years. We illustrate this using real-world data from GENVASC and the initiation of statin treatment in the additional individuals identified as being at high risk (see Supplementary Text). If all additional subjects identified as being at high risk were started on a statin our modelling shows that in all ages an extra 5.9 (95% CI: 5.3–6.4) cardiovascular events per 100 000 patient-years would be potentially preventable by using the combination of QRISK2 and IRT over QRISK2 alone while in the 40–54 years age, an extra 5.6 (95% CI: 5.0–6.4) cases per 100 000 patient-years could be prevented (see Supplementary Text). However, in GENVASC (see Supplementary Text) and in the wider NHSHC programme^33^ only about a quarter of individuals identified as high risk are currently prescribed a statin. The findings emphasise the importance of coupling the better identification of those at increased risk with the addition of a CVD-PRS with better application of preventative measures to obtain the greatest clinical impact.

Our study has evaluated the incremental gain from adding a CVD-PRS to the current clinical score-based risk assessment, where we believe its first application will be in clinical practice. However, a CVD-PRS could also be used to identify individuals at a high genetic risk independent of their clinical score-based risk and this could be done at a much earlier age when the clinical scores are less discriminating. Whether identifying such individuals and targeting preventative measures towards them would be acceptable to such individuals and bring substantial clinical benefit requires further research.

Genome-wide arrays now cost less than many routine clinical blood tests, and our findings provide evidence that the addition of a CVD-PRS into the assessment of CVD risk could lead to substantial clinical benefit. However, its integration into clinical practice, like any new test, will amongst other things require a detailed economic analysis, a mechanism to update the PRS calculation as new genetic information emerges especially in non-white ethnicities, and further implementation studies such as HEART^34^ in order to assess the quality control of the genotyping assay, standardisation of the CVD-PRS, and feasibility, acceptance and support by clinicians and patients.

## Supplementary Material

ehae342_Supplementary_Data

## Data Availability

Data are available on request.
