# Supplementary material for: Polygenic risk score adds to a clinical risk score in the prediction of cardiovascular disease in a clinical setting
Source: Eur Heart J. 2024 Jun 7;45(34):3152–60. doi: 10.1093/eurheartj/ehae342 (PMC11379490; doi:10.1093/eurheartj/ehae342)
Supplement: ehae342_Supplementary_Data [file ehae342_supplementary_data.docx]

**Supplementary Tables, Figures and Text: Polygenic risk score adds to a clinical risk score in the prediction of cardiovascular disease in a clinical setting**

Nilesh J Samani et al.

**Supplementary Table 1.** Sociodemographic characteristics of GENVASC study participants compared to a large population of NHSHC attendees across England

|  | NHSHC data*  n=5,102,758 | GENVASC study  n=44,141 |
| --- | --- | --- |
|  |  |  |
| Male Gender | 2 311 604 (45.3) | 19,569 (44.3) |
| **Age bands (years)**  40-49  50-59  60-74 |  |  |
|  | 1,951,264 (38.2) | 17,795 (40.3) |
|  | 1,742,003 (34.1) | 13,068 (29.6) |
|  | 1,409,491 (27.7) | 13,277 (30.1) |
| Missing |  | 1 |
| **Ethnicity** |  |  |
| White | 4,067,864 (79.7) | 36,704 (83.2) |
| Asian | 368,145 (7.2) | 5,265 (11.9) |
| Black | 148,160 (2.9) | 842 (1.9) |
| Other ethnicity | 142,621 (2.8) | 904 (2.0) |
| Missing data | 375,968 (7.4) | 426 (1.0) |
| **Townsend Deprivation Score** |  |  |
| 1st quintile (least deprived) | 1,129,670 (22.1) | 13,768 (31.2) |
| 2nd quintile | 1,094,925 (21.5) | 9,549 (21.6) |
| 3rd quintile | 1,027,096 (20.1) | 6,961 (15.8) |
| 4th quintile | 954,656 (18.7) | 5,283 (12.0) |
| 5th quintile (most deprived) | 893,194 (17.5) | 7,168 (16.2) |
| Missing data | 3217 (0.1) | 1,412 (3.2) |

Data are presented as n (percentage). *Sociodemographic characteristics of attendees of NHS Health Check across England for years 2012-2017 reproduced from Patel R et al.^1^ The Townsend Deprivation Score is a composite measure of material deprivation of the population in UK and is published by the UK Office of National Statistics. The score is calculated using four census variables for any given geographical area using postal codes. These variables include: unemployment - defined as the percentage of economically active residents who are unemployed; non-car ownership measuring the number of households without a car; non-home ownership measuring the number of households renting and owning their accommodations; overcrowding – measuring whether the accommodation is suitable in size for the number of occupants. The Townsend Deprivation Scores are calculated using percentages of the weighted and normalised (where appropriate) four previously listed indicators. Townsend Deprivation Scores are usually split into quintiles to provide map visualisations of deprivation and to assess the spread of deprivation across areas. https://statistics.ukdataservice.ac.uk/dataset/2011-uk-townsend-deprivation-scores

**Supplementary Figure 1.** Percentage of patients having NHSHCs recruited into GENVASC


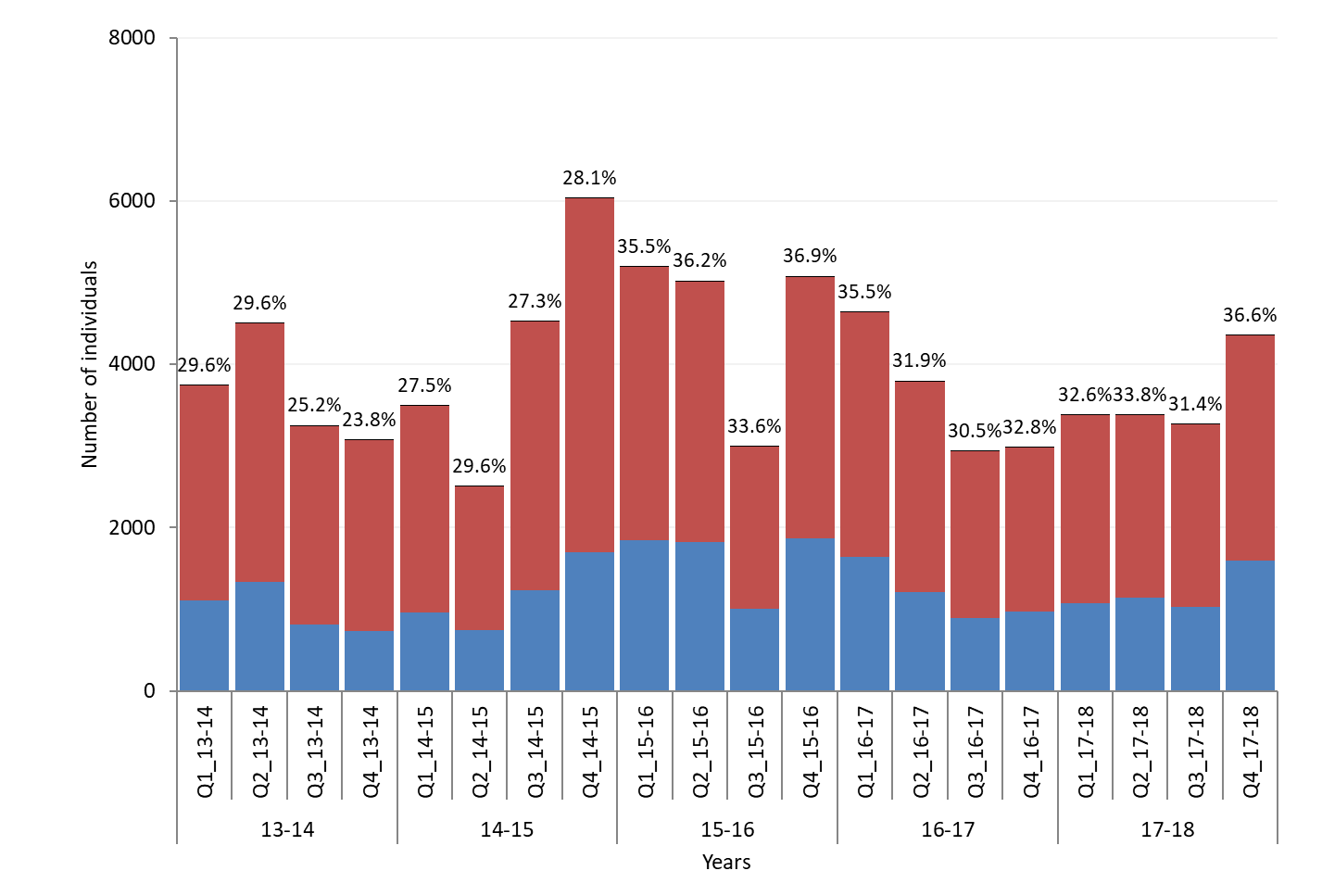


Quarter-by quarter data for the percentage of individuals having a NHSHC in participating practices in Leicester (red) who were recruited into GENVASC (blue bar) for the calendar years 2013-14 to 2017-2018. The total number of actively recruiting practices varied from 27 to 122 and the total number of NHSHC varied each year depending on local health priorities. Data are not available for 2012 and 2019-2020.

**Supplementary Figure 2:** Percentage of female and non-white ethnicity cases identified as at high risk by QRISK2 and a combination of QRISK2 and IRT


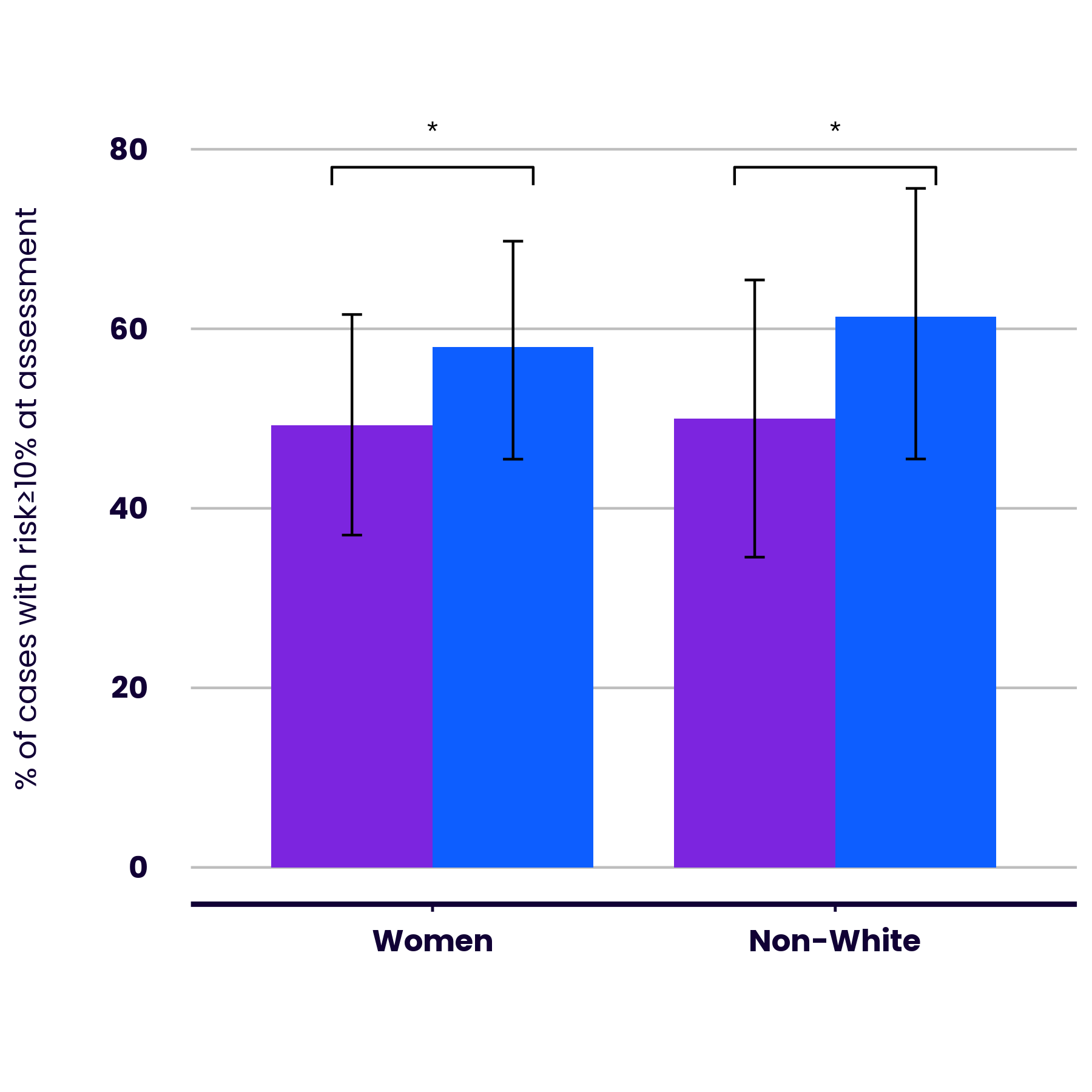


Y-axis represents the percentage of all major CVD event cases identified as high risk by QRISK (purple) or by a combination of QRISK2 and IRT (blue) at the time of their NHSHC assessment for female cases (left hand panel) and non-white ethnicity cases (right hand panel). * indicates that the p-value for difference is <0.05.

**Modelling the potential clinical impact of the additional cases at high risk identified by IRT.**

To illustrate the potential clinical impact of adding an IRT for CVD risk assessment, we modelled the benefit from statin treatment initiation in preventing major CVD events in the additional number of subjects identified as high risk by a combination of QRISK2 and IRT compared with QRISK2 alone, again using real world data from GENVASC. We identified the number of new cases of major CVD outcomes in the entire GENVASC cohort not taking a statin at the time of their NHSHC until January 2023, and calculated the incidence rate per 100,000 person years follow-up. As this incidence rate may be affected by statin treatment initiated after the NHSHC, we uplifted it based on the observed 26% statin initiation rate in subjects identified at high risk in GENVASC, and a predicted 25% reduction in major CVD events through statin treatment seen in clinical trials.^2^ Then, based on the difference in the proportion of subjects identified as at high risk by QRISK2 alone and the combination of QRISK2 and IRT in the nested case-control study, we estimated the absolute number of additional major CVD events that could be potentially prevented from the 26% rate of statin initiation observed in GENVASC in such subjects. To assess the full potential, we extrapolated these data to assess the additional gains in CVD event prevention for different rates of statin prescription in those identified as at increased risk.

Up to January 2023, with a median follow-up 6.5 years, amongst individuals not already on a statin at the time of their NHSHC (n=42,214) in GENVASC, there were 681 major CVD events. This equates to an incident rate of 275.0 (95% CI: 255.1-296.5) cases per 100,000 follow-up years, uplifted to 292.9 (95% CI: 27.7-315.8) cases per 100,000 follow-up years taking into account the observed statin prescription rate after the NHSHC in GENVASC and the predicted reduction in CVD events by statins (see above). QRISK2 would have identified 169.3 (95% CI 157.1-182.5, 57.8%) of these cases as at high risk at the NHSHC while the combination of QRISK2 and IRT would have identified 193.0 (95% CI: 199.0-208.1, 65.9%). Based on a statin initiation of 26% in such cases and a subsequent reduction of CVD events of 25% amongst those taking statins, 11.0 (95% CI: 10.2-11.9) cases per 100,000 years follow-up would have been prevented through the application of QRISK2. With the addition of IRT, the number of cases prevented would be 12.6 (95% CI: 11.6-13.5), an uplift of 14.6%. In individuals between 40-54 years of age, the adjusted incident rate observed was 180.6 (95% CI: 150.6-204.4) cases per 100,000 patient years. QRISK2 alone would have identified 45.2 (95% CI 39.9-51.1, 25.0%) of these while the combination of QRISK2 and IRT would have identified 67.7 (59.8-76.4, 37.5%). In the 40-54 years age-group, statin initiation after the NHSHC in those at high risk was 34%. Using the same proportionate benefit from statins, QRISK2 would have prevented 3.8 (95% CI: 3.4-4.3) cases per 100,000 years follow-up while the combination of QRISK2 and IRT would have prevented 5.8 (95%CI: 5.1-6.5) cases per 100,000 years follow-up, an uplift 52.6%. Although the relative increase in potential number of preventable cases by a combination of QRISK2 and IRT over QRISK2 alone would be the same across different rates of statin initiation, the absolute number of extra cases prevented would vary depending on statin usage as overall more cases would be prevented with higher use (**Supplementary** **Figure 3**). With 100% statin use, in all ages an extra 5.9 (95% CI: 5.3-6.4) cases per 100,000 patient years would be potentially preventable by using the combination of QRISK2 and IRT over QRISK2 alone while in the 40-54 years age an extra 5.6 (95% CI: 5.0-6.4) cases per 100,000 patient years would be prevented (**Supplementary Figure 3**).

**Supplementary Figure 3:** Estimated reduction of major CVD events from initiation of statin treatment in those identified as high risk by QRISK2 or by a combination of QRISK2 and IRT


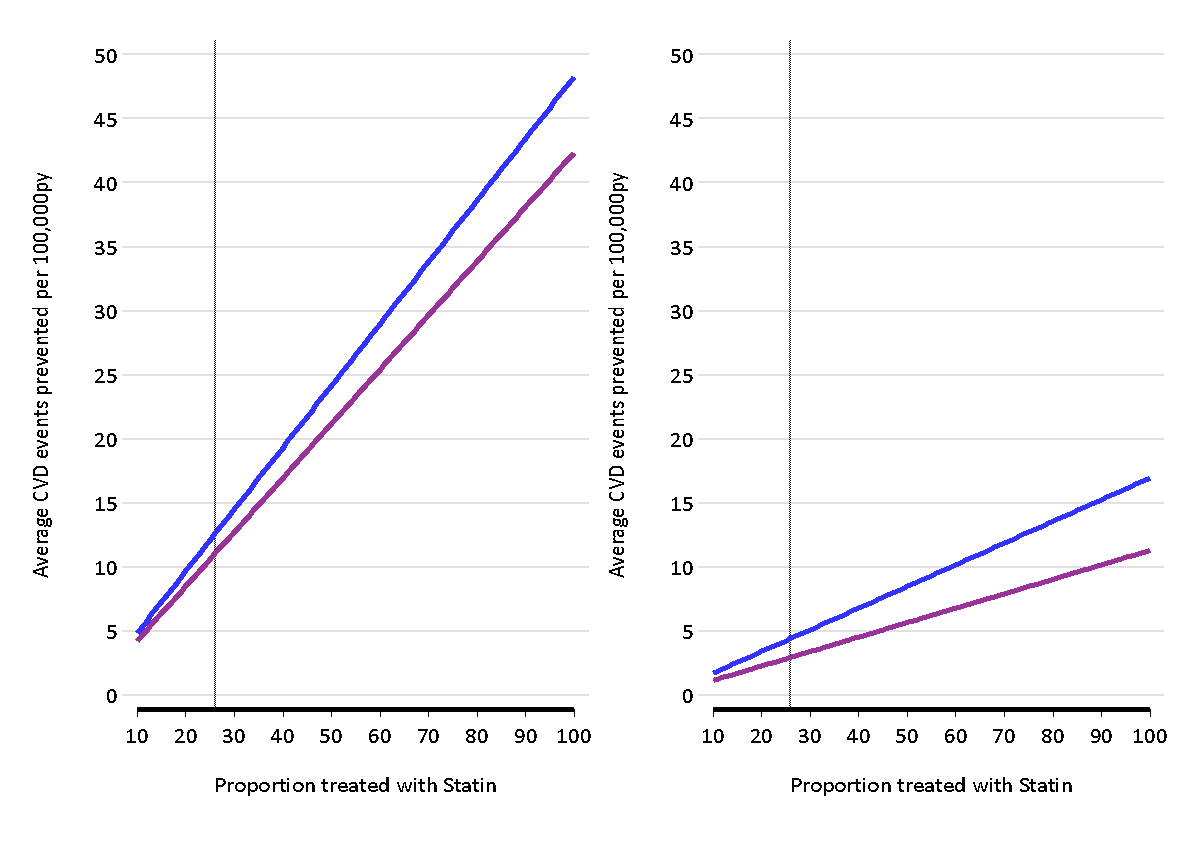


The average number of major CVD events that could potentially be prevented per 100,000 person years from different rates of statin initiation in those identified as high risk through the use of QRISK2 (purple) or a combination of QRISK2 and IRT (blue) for risk assessment. The left panel shows the modelling for all individuals over 40 years and the right panel for individuals aged 40-54 years. Vertical grey lines represent observed statin initiation rates in GENVASC in those found to be at high risk in each group.

**Supplementary References**

1. Patel R, Barnard S, Thompson K, Lagord C, Clegg E, Worrall R, et al. Evaluation of the uptake and delivery of the NHS Health Check programme in England, using primary care data from 9.5 million people: a cross-sectional study. *BMJ Open* 2020;10:e042963. doi: https://doi.org/10.1136/bmjopen-2020-042963

2. Yebyo HG, Aschmann HG, Kaufmann M, Puhan MA. Comparative effectiveness and safety of statins as a class and of specific statins for primary prevention of cardiovascular disease: A systematic review, meta-analysis, and network meta-analysis of randomized trials with 94,283 participants. *Am Heart J* 2019;210:18-28. doi: https://doi.org/10.1016/j.ahj.2018.12.007
